# Effects of Pregnancy on Liver and Kidney Cyst Growth Rates in Autosomal Dominant Polycystic Kidney Disease: A Pilot Study

**DOI:** 10.3390/jcm14113688

**Published:** 2025-05-24

**Authors:** Vahid Bazojoo, Vahid Davoudi, Jon D. Blumenfeld, Chenglin Zhu, Line Malha, Grace C. Lo, James M. Chevalier, Daniil Shimonov, Arman Sharbatdaran, Hreedi Dev, Syed I. Raza, Zhongxiu Hu, Xinzi He, Arindam RoyChoudhury, Martin R. Prince

**Affiliations:** 1 Department of Radiology, Weill Cornell Medicine, New York, NY 10022, USA; vab4003@med.cornell.edu (V.B.); vad4005@med.cornell.edu (V.D.); chz4009@med.cornell.edu (C.Z.); gcl9003@med.cornell.edu (G.C.L.); ars4017@med.cornell.edu (A.S.); hrd2001@med.cornell.edu (H.D.); zsh4001@med.cornell.edu (Z.H.); xih4004@med.cornell.edu (X.H.); 2 Department of Medicine, Division of Nephrology and Hypertension, Weill Cornell Medical College, New York, NY 10021, USA; jdblume@nyp.org (J.D.B.); lim9120@med.cornell.edu (L.M.); jac9014@nyp.org (J.M.C.); das2041@nyp.org (D.S.); 3 The Rogosin Institute, New York, NY 10021, USA; 4 Department of Internal Medicine, Detroit Medical Center, Detroit, MI 48201, USA; syedimranraza@texashealth.org; 5 Division of Biostatistics, Department of Population Health Sciences, Weill Cornell Medicine, New York, NY 10065, USA; arr2014@med.cornell.edu

**Keywords:** ADPKD, pregnancy, liver, kidney, cysts, estrogen

## Abstract

**Background/Objectives**: Polycystic liver disease (PLD) is the most common extrarenal manifestation of autosomal dominant polycystic kidney disease (ADPKD). PLD is more prevalent in women, and women have larger liver cysts, possibly due to estrogen-related mechanisms. Maternal estrogen levels normally increase during pregnancy. Thus, we investigated the pregnancy-associated increase in liver volume, liver cyst volume, total kidney volume (TKV), and kidney cyst growth rates in ADPKD patients. **Methods**: Kidney, liver, and cyst volumes were measured in 16 ADPKD patients by magnetic resonance imaging (MRI) at multiple timepoints before and after pregnancy. The log-transformed TKV, liver volume, and cyst volume growth rates during a period with pregnancy were compared to a period without pregnancy. **Results**: In ADPKD patients, a higher annualized liver cyst growth rate was observed during a period with pregnancy compared to a period without pregnancy (34 ± 16%/yr vs. 23 ± 17%/yr; *p*-value = 0.005). Liver volume growth was also higher during a period with pregnancy, 6 [2, 7]%/yr vs. 0.3 [−0.4, 2]%/yr (*p*-value = 0.04). In addition, the mean kidney cyst growth rate was higher (12 ± 11%/yr vs. 4 ± 9%/yr; *p*-value = 0.05), and there was a trend toward a pregnancy-associated increase in the TKV growth rate (6 [4, 8]%/yr vs. 3 [0.8, 5]%/yr, (*p*-value = 0.14) during a period with pregnancy. **Conclusions**: In patients with ADPKD, the liver volume and cyst volume growth rates increased during pregnancy. This supports the hypothesis that the estrogen-mediated stimulation of liver cyst growth may contribute to the severe polycystic liver disease that is more prevalent in women than men with ADPKD. Further studies with larger populations are needed to explore the mechanisms and long-term implications of these findings.

## 1. Introduction

Autosomal dominant polycystic kidney disease (ADPKD) is the most common inherited cause of end-stage kidney disease (ESKD) in the United States [1]. ADPKD is caused by mutations in the *PKD1* or *PKD2* genes [2,3], and rarely by mutations in other genes (e.g., *GANAB* [4], *DNAJB11* [5], *IFT140* [6], and *ALG9* [7]). Extrarenal manifestations of ADPKD include polycystic liver disease (PLD) and cysts in the pancreas, prostate, and arachnoid spaces.

Liver cysts occur in more than 90% of ADPKD patients by the age of 35 years [4] and are larger, more numerous, and occur earlier in women [8,9]. The increased liver cyst burden in female ADPKD patients has been hypothesized to be related to female steroid hormones, particularly estrogen [10]. It is known that liver is an estrogen-sensitive organ, where hepatocytes express estrogen receptors [11]. In healthy pregnant women, the liver volume has been observed to increase by 15% from early to late pregnancy [12], supporting the role of estrogen in hepatic tissue growth. For females with ADPKD, this hormonal sensitivity may contribute to more pronounced liver enlargement due to additional hepatic cyst growth. Therapeutic strategies targeting estrogen pathways are being explored as a promising approach to treat polycystic liver disease [10]. For example, a case report described a 59-year-old woman with polycystic liver disease who experienced a significant reduction in cyst volume following treatment with tamoxifen, a selective estrogen receptor antagonist [13]. Although liver function remains preserved in most patients, the mass effect of large liver cysts can lead to significant morbidity, including abdominal discomfort, gastric confinement and early satiety. Additionally, the resulting protruding abdomen may negatively impact body image, psychological well-being [14] and decrease life quality [15].

Given the potential for estrogen to accelerate liver cyst growth, female ADPKD patients with liver involvement are counseled to be cautious with estrogen exposure, including hormonal contraception, pregnancy, and postmenopausal hormone replacement therapy. According to the 2025 KDIGO clinical practice guideline on ADPKD, individuals without or with only mild polycystic liver disease may use low-estrogen contraceptives [16]. However, there remains a significant gap in guidelines concerning pregnancy in women with ADPKD. Evaluating the effects of pregnancy on the progression of liver and kidney cysts is essential to support informed reproductive decision-making in this patient population.

In the current study, we evaluated the total kidney volume (TKV) and liver volume growth rates in ADPKD, using patients as their own controls, by measuring liver and kidney growth rates during periods with pregnancy and during periods without pregnancy. We also examined the effects of pregnancy on the cyst growth rate.

## 2. Materials and Methods

### 2.1. Subjects and Data Extraction

This Health Insurance Portability and Accountability Act (HIPAA) compliant study was approved by the Weill Cornell Medicine Institutional Review Board. Most of the ADPKD subjects were enrolled in the Rogosin Institute PKD Data Repository (NCT00792155) and signed informed consent. A retrospective review of existing clinical data for non-ADPKD control patients and ADPKD patients not enrolled in the Rogosin PKD Data Repository was approved by the Institutional Review Board at Weill Cornell with a waiver of the consent (Study IRB approval numbers: 0304006105 and 1610017623).

The inclusion criteria were as follows: (i) patients with a diagnosis of ADPKD based upon Pei Ravine criteria [17]; (ii) patients with at least three serial abdominal MRIs performed before and after pregnancy, and before and after a period of not being pregnant; and (iii) patients with available documentation of pregnancy history. The exclusion criteria were (i) patients undergoing estrogen therapy; (ii) patients treated with somatostatin analogs or other pharmaceuticals that could affect liver or kidney volume (e.g., tolvaptan); (iii) patients undergoing procedures that reduce cyst size or number in the liver or kidneys; (iv) patients in which it was impossible to measure the kidney and liver volume due to artifacts or the incomplete inclusion of the organ within the field-of-view; (v) patients with missing clinical data regarding pregnancy history; and (vi) male subjects.

The patients’ demographic, clinical, genetic, and laboratory data were extracted from the electronic medical records. Images were acquired from the Radiology Picture Archiving and Communication System (PACS).

### 2.2. MRI Acquisition

The kidneys and liver were scanned together in a single sequence during a single breath hold, when feasible, at 1.5 or 3 T. If multiple breath holds were required to acquire all slices, patients were instructed to hold their breath similarly for each breath hold. If the kidneys or liver were too large to be imaged in one scan, two separate scans (upper and lower) were acquired for each organ and then combined into a single set of images using the compose or add/subtract function. Pulse sequences included axial/coronal T2-weighted single-shot fast spin echo (SSFSE) images, 3D spoiled gradient-recalled echo T1-weighted images with fat suppression, and steady-state free precession (SSFP).

### 2.3. Image Analysis and Calculation of Growth Rates

A 3D multimodality deep learning model (www.TraceOrg.com) provided initial kidney and liver segmentations [18] that were manually corrected independently by two observers (VB, VD) to assess the inter-observer measurement variability on every axial and coronal T1, T2, and SSFP image using ITK-SNAP software, version 4.2.0 (Penn Image Computing and Science Laboratory, Philadelphia, PA, USA). The measurements made from the multiple pulse sequences in each MRI exam were averaged. Cyst volumes were only measured on T2-weighted images. After the initial cyst labeling was performed by the deep learning model, manual corrections of the labeling were made independently by the same two experts.

Each available pair of consecutive MRI scans provided an opportunity to calculate the organ and cyst volume growth rates during the intervening period. Assuming exponential growth, the TKV, total kidney cyst volume, liver volume, and total liver cyst volume growth rates were calculated as $r=\frac{1}{t}\times \ln\;(\frac{N_{t}}{N_{0}})$, where *t* is the time over which the change occurs, *N_t_* is the volume at time *t*, and *N*_0_ is the initial volume at time *t* = 0. Periods between consecutive MRI scans that included 1 or more pregnancies were defined as a “period including pregnancy”. Periods between consecutive MRI scans that did not include pregnancy were defined as “period not including pregnancy”. To compare the periods during pregnancy to those not during pregnancy within individual subjects, the average growth rate during all periods with pregnancy was compared to the average growth rate during all periods without pregnancy.

### 2.4. Effect of Gravida

In order to explore the effect of gravida, a subgroup analysis was performed comparing the mean rate of liver, kidney, renal cyst and liver cyst growth for the 1st, 2nd and 3rd + later pregnancies. In addition, the mean organ/cyst growth rates were calculated for the periods not including pregnancy occurring prior to the 1st pregnancy, after 1st pregnancy, after 2nd pregnancy and after 3 or more pregnancies to determine if having multiple pregnancies had a cumulative effect lasting into a period without pregnancy beyond the last pregnancy.

### 2.5. Effect of In Vitro Fertilization (IVF)

To assess the effect of IVF on the liver, liver cyst, kidney and kidney cyst growth rates, patients with IVF cycles (where the period of IVF was during the period including pregnancy) were compared to ADPKD patients with pregnancies conceived naturally.

### 2.6. Comparison to Pregnancies in Women Without ADPKD

Since it is known that pregnancy can affect organ volume [12], a control population of pregnant women without ADPKD was matched by age (year of birth) in order to determine how much any change in the liver and kidney volumes was related to underlying pregnancy as opposed to liver and renal cyst growth.

### 2.7. Statistical Analysis

The mean and standard deviation (SD) were reported for normally distributed data; median and interquartile ranges (IQR; Q1 and Q3) were provided for non-normally distributed continuous variables. Shapiro’s test and histograms were used to determine the normality of the data.

The paired or independent *t*-test was used to compare means between two groups when the data had a normal distribution; the Mann–Whitney U test was used for data with distributions that were not normal. We conducted one-way analysis of variance (ANOVA) to compare means between more than two groups for data that met the normality and homogeneity of variances assumptions. If we found a significant ANOVA, we followed up with a post hoc comparison with Tukey’s HSD test. For data that did not meet the normality assumption or if the homogeneity of variance assumption was violated, we conducted a Kruskal–Wallis H test (non-parametric alternative to ANOVA) to compare group medians. Then, we conducted post hoc pairwise comparisons with a Dunn–Bonferroni test. Chi-square was applied to determine the statistical significance of categorical variables in the analysis. The correlation was assessed using Pearson’s correlation and Spearman’s correlation analysis for linear relationships in normal and non-normal distributions, respectively. A *p*-value of < 0.05 was considered statistically significant.

## 3. Results

### 3.1. Demographic Data

**Figure 1:**
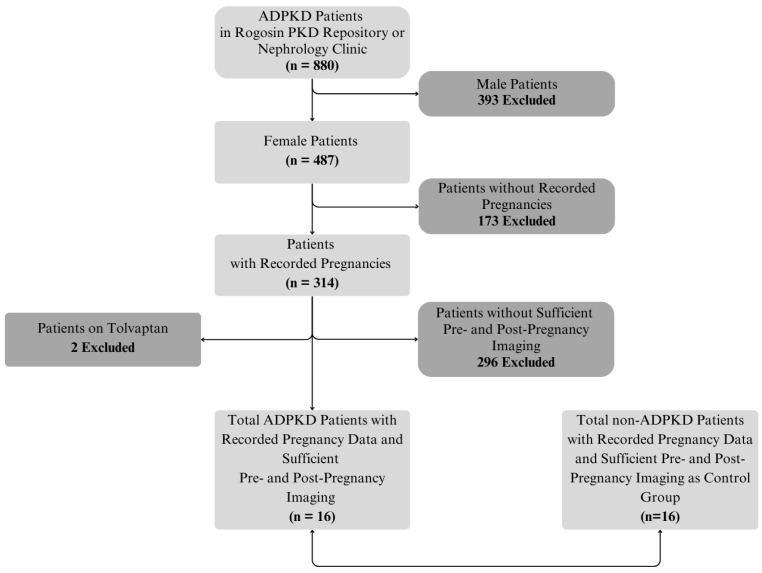
Patient flow chart showing 16 ADPKD and 16 matched non-ADPKD patients.

Sixteen ADPKD subjects (38 pregnancies, median gravida = 2) met the inclusion/exclusion criteria and were included in this analysis (Figure 1). The demographic data for these 16 ADPKD subjects and 16 date-of-birth matched controls without ADPKD (34 pregnancies, median gravida = 2) are shown in Table 1. Nine (56%) ADPKD subjects and 2 controls underwent in vitro fertilization (IVF). Two subjects had IVF prior to any MRI scans. Underlying disorders in these control subjects included Crohn’s diseases (*n* = 7), irritable bowel syndrome (*n* = 1), vertebral hemangioma (*n* = 1), chronic pancreatitis (*n* = 2), and focal nodular hyperplasia (*n* = 5).

### 3.2. Interobserver Agreement

There was excellent agreement between the two observers in measuring organ volumes, with ICC values of 0.97 and 0.99 for the kidney and liver volume, respectively, and ICC values of 0.98 and 0.99 for the kidney cyst and liver cyst volume, respectively.

### 3.3. Effect of Pregnancy on Liver, Kidney, Cyst Growth

**Figure 2:**
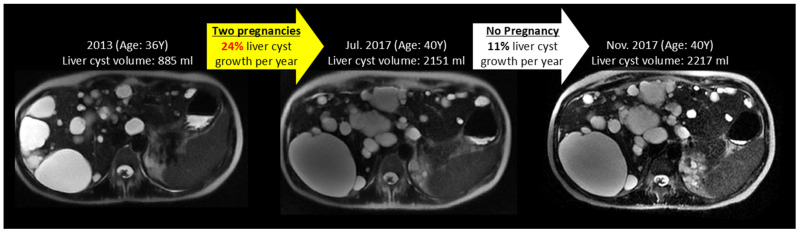
This female ADPKD patient with the *PKD1* gene mutation had a 24% per year liver cyst growth rate during her fifth and sixth pregnancies (yellow arrow) and an 11% per year liver cyst growth rate during a period not including pregnancy (white arrow). The hepatic cyst volume more than doubles between the left image, prior to her fifth pregnancy, and the middle image, 4 years later after her sixth pregnancy, showing an annual growth rate of 24%. But the hepatic cysts grew minimally between the middle image and right image, a period without pregnancy, with an 11% annual growth rate.

**Figure 3:**
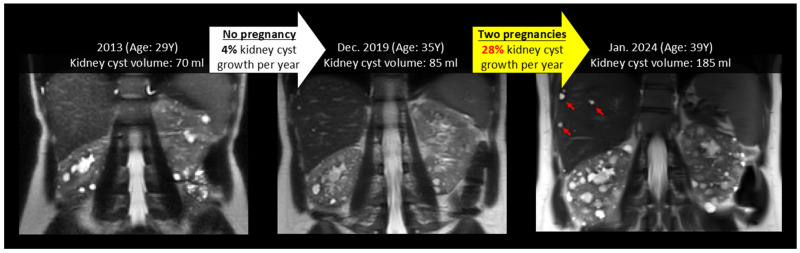
This female ADPKD patient with the *PKD1* gene mutation had a 4% annual kidney cyst growth rate during a period not including pregnancy (white arrow) and a 28% annual kidney cyst growth rate during a period including two pregnancies (yellow arrow). Note also the rapid growth of liver cysts (red arrowheads) during a period including pregnancy, which increased from 2 mL to 13 mL from 2013 to 2019 (without pregnancy), compared with 13 mL to 55 mL from 2019 to 2014 (including two pregnancies).

**Figure 4:**
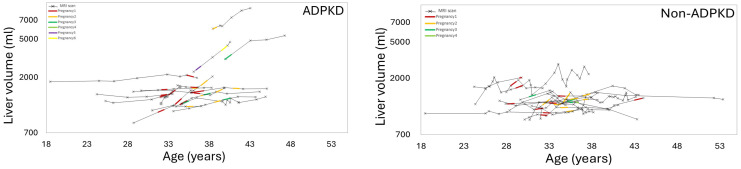
Liver volume versus patient age, showing the liver growth during periods including pregnancy (colored line segments) compared to the periods without pregnancy (black line segments) in ADPKD mothers (left) and mothers without APDKD (right). Notice that the ADPKD mothers experienced greater liver volume growth compared to non-ADPKD mothers. Also note that during the peak childbearing years, from age 30 to 40, the group of 16 ADPKD mothers had greater liver volume growth compared to ages <30 and >40 years, showing that pregnancy is associated with increased liver growth. Non-ADPKD patients (right) had comparatively less liver growth during pregnancy.

**Figure 5:**
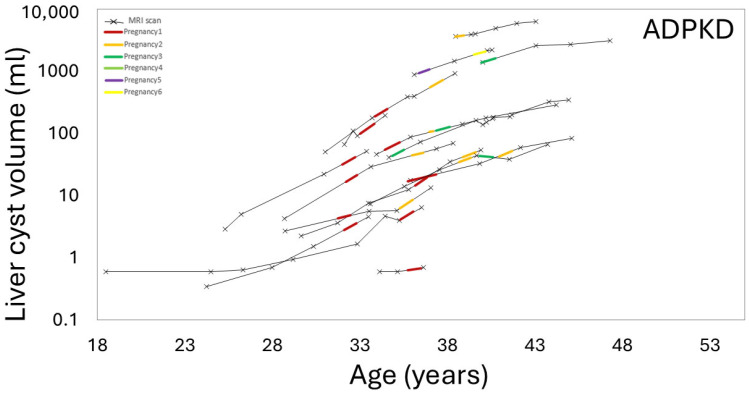
Liver cyst volume versus patient age, showing an increased liver cyst growth rate (ages 30 to 40 years) during periods including pregnancy (colored line segments) compared to periods without pregnancy (black line segments) in ADPKD subjects.

Figure 2 and Figure 3 show examples of ADPKD subjects in which liver and kidney cysts grew faster during a period with pregnancy compared to the non-pregnancy period. Quantitative analysis confirmed that the liver volume growth rate during a period with pregnancy was higher compared to the liver volume growth rate during the non-pregnancy periods (*p*-value = 0.04) (Table 2, Figure 4, Supplemental Tables S1 and S2). The liver cyst growth rate was also greater during the period with pregnancy compared to the non-pregnancy period (34 ± 16%/yr vs. 23 ± 17%/yr; *p*-value = 0.005) (Figure 5).

**Figure 6:**
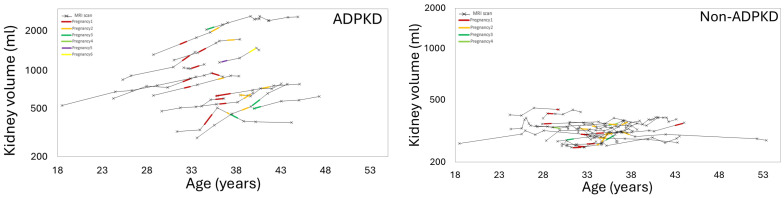
Total kidney volume versus age, showing how the kidney volume grows during periods including pregnancy (colored line segments) compared to the non-pregnancy period (black line segments) in ADPKD subjects (left); this is greater than for non-ADPKD subjects (right).

**Figure 7:**
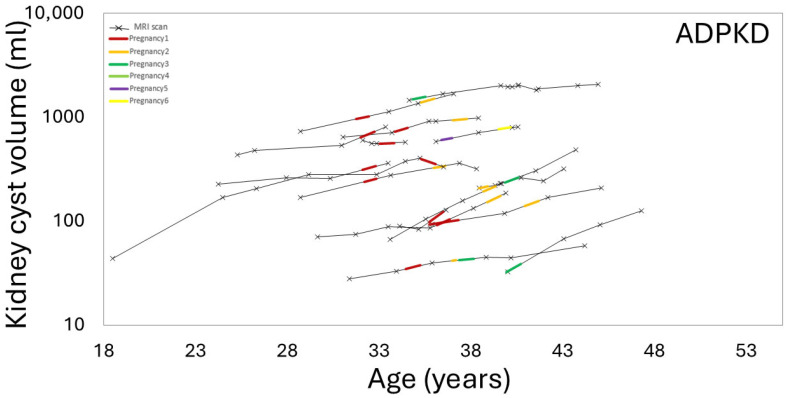
The growth rate of kidney cysts increased during periods including pregnancy (colored line segments), between the ages of 30 and 40, compared to non-pregnancy periods (black line segments) in ADPKD female subjects.

There was a trend toward greater TKV growth rates during the period including pregnancy compared to during the non-pregnancy period, (6 [4, 8]%/yr vs. 3 [0.8, 5]; *p*-value = 0.14) (Figure 6). Furthermore, the mean kidney cyst growth rate was greater during the period including pregnancy compared to the non-pregnancy period (12 ± 11%/yr vs. 4 ± 9%/yr, *p*-value = 0.05) (Figure 7).

Additional comparisons between patients with increased liver cyst and kidney cyst growth during pregnancy and those without increased cyst growth during pregnancy are shown in Supplemental Tables S3 and S4.

### 3.4. Effect of Gravida and Cumulative Pregnancy Effect on Kidney, Liver, Cysts Growth Rates

We did not find significant differences in the pregnancy order for liver, liver cyst, kidney, and kidney cyst growth in ADPKD subjects (Table 3). However, there was a non-significant trend towards reduced liver cyst growth with higher gravida (*p*-value = 0.11). To further explore the potential cumulative impact of multiple pregnancies, we analyzed the growth rates during non-pregnancy intervals, stratified by pregnancy history (Table 4). There was a trend toward lower liver cyst and TKV growth rates with an increasing number of prior pregnancies, although these differences were not statistically significant. These trends may suggest that individuals who experience rapid liver cyst growth during their first pregnancies are less likely to pursue additional pregnancies.

### 3.5. Effect of IVF on Liver, Kidney, and Cyst Growth Rates

Given the increased estrogen exposure associated with IVF, a subgroup analysis of naturally conceived versus IVF-induced pregnancies was performed (Table 5). However, no statistically significant differences were observed in the annual growth rates of the liver volume, liver cyst volume, TKV, or kidney cyst volume with IVF.

### 3.6. Effect of Pregnancy on Organ Growth in Non-ADPKD Subjects

Sixteen age-matched non-ADPKD control subjects were included in the analysis (Table 1). Compared to ADPKD subjects, the control participants were younger at the time of their first pregnancy (31 vs. 35 years, *p*-value = 0.04), had a lower rate of IVF use (12% vs. 56%), and had shorter intervals between MRI examinations (330 vs. 693 days) and during the period including pregnancy (810 vs. 715), which was considered not likely to impact this analysis. In control subjects, the annual liver volume growth rate was slightly higher during pregnancy periods compared to non-pregnancy periods (3% vs. 1% per year). However, there were no significant differences in the TKV growth rates between the pregnancy and non-pregnancy periods (Table 6).

## 4. Discussion

We evaluated serial MRI scans prior to and after pregnancies to determine the effect of pregnancy on the kidney and liver volume growth rates in 16 women with ADPKD. We found that the liver volume growth rate was higher during a period including pregnancy compared to a period when these patients were not pregnant. By measuring cyst volumes, we were able to confirm that higher liver volume growth rates during a period including pregnancy in ADPKD reflected, at least partly, increases in liver cyst volume growth rates. In addition, kidney cyst volumes grew faster during a period including pregnancy and there was a trend toward faster TKV growth rate during pregnancy.

The impact of pregnancy on the progression of polycystic liver disease (PLD) in patients with ADPKD has long been a topic of scientific interest. As early as the 1990s, there were reported associations between pregnancy and an increase in the number, size, and prevalence of liver cysts in women with ADPKD [19,20]. The reported acceleration of hepatic cyst growth during pregnancy has been hypothesized to result from the promotion of cystogenesis by the female steroid hormone. Supporting this hypothesis are several clinical observations: (1) a marked increase in liver cyst volume among women aged 25–34 years, potentially due to the combined effects of pregnancy and oral contraceptive use [8]; (2) a significant reduction in liver volume among postmenopausal women over 48 years with severe PLD [21,22]; and (3) hepatic cyst enlargement in postmenopausal women receiving hormone replacement therapy [23]. However, more recent studies that retrospectively assess the impact of hormonal exposure with more advanced organ and cyst measurements have challenged those observations. Hogan et al. [24] found no significant differences in the prevalence or volume of liver cysts between parous and nulliparous women after adjusting for age. Similarly, Bae et al. [22] reported no differences in the liver cyst or liver volume growth rates between hormonal birth control users and non-users among female ADPKD patients, and reported similar liver and liver cyst growth rates.

Beyond hepatic effects, physiological changes during pregnancy, such as the activation of the renin–angiotensin–aldosterone system, elevated circulating vasopressin and increased renal blood flow, may also contribute to accelerated kidney cyst formation and growth and TKV growth [25,26]. In support of this, Zheng et al. [27] observed a more rapid increase in height-adjusted TKV (Ht-TKV) following a first pregnancy in women with PKD compared to their non-pregnant counterparts. However, this study was limited by its small cohort size (*n* = 6 per group) and reliance on ultrasound-based ellipsoid volume estimates.

These results are important for female ADPKD patients as they allow us to understand the potential magnitude and consequences of changes in cyst growth during pregnancy. Although there was an increase in the liver cyst growth rate during pregnancy compared to when they were not pregnant, the total amount of cyst growth was small for all the women in this study. This reflected their low hepatic cyst burden prior to pregnancy. Thus, ADPKD patients can make an informed choice about the added risk of pregnancy when experiencing increased liver and kidney cyst growth. Furthermore, since there was a wide variation in the liver cyst growth rate among the ADPKD patients in this study, it might make sense for women to assess their liver, kidney and cyst growth rates via serial MRI before and during pregnancy in order to make informed choices about the risks of additional pregnancies.

A major strength of our study was the utilization of advanced imaging modalities, which measured both the volume of cysts and overall organ volumes to track changes in the liver and kidneys over time with precision. These enabled us to serially capture the dynamic nature of cyst growth and organ enlargement in the context of one or more pregnancies. This approach enabled us to detect subtle changes that might not have been detectable in earlier studies.

Our study has several limitations. The retrospective collection of data may have biased the study toward patients more likely to undergo imaging, although most subjects were enrolled in a PKD research repository that required routine biennial imaging to minimize this bias. The small sample size limited the statistical power and our ability to simultaneously evaluate multiple parameters with multivariate regression. These findings could be used to generate a hypothesis and be confirmed in future prospective studies with more subjects. The “period including pregnancy” in this study occurred over a median of 2 years, which may have obscured the effect of pregnancy on cyst and organ volume growth. Some ADPKD subjects reported a history of IVF, but a subgroup analysis of naturally conceived versus IVF conceived pregnancies did not find that IVF had any effect. Since our patients did not have giant livers (median volume pre-pregnancy was 1594cc compared to 1166cc in non-APDKD controls), these data may not be applicable to those rare ADPKD patients with more severe PLD.

## 5. Summary and Conclusions

Our findings in this longitudinal observational study of a small sample of ADPKD patients suggest that pregnancy is associated with increased liver cyst and kidney cyst growth rates. However, these increases were small and did not occur in all patients with ADPKD. This study was not designed to evaluate the pathophysiologic basis for this phenotypic heterogeneity, but the results support an association with pregnancy. Further studies with larger populations are needed to explore the mechanisms and long-term implications of these findings.

## Figures and Tables

**Table 1:** Demographic data, laboratory data, and MRI exam intervals in ADPKD subjects (*n* = 16) and non-ADPKD control subjects (*n* = 16).

	ADPKD (*n* = 16)	Non-ADPKD (*n* = 16)	*p*-Value
Age at the latest scan (yr)	40 ± 5	39 ± 5	0.78
Age at the first pregnancy (yr)	35 ± 2	31 ± 5	0.04
Race			0.45
White	12 (75%)	10 (63%)	
Asian	3 (19%)	2 (12%)	
Others or Declined	1 (6%)	4 (25%)	
Height (m)	1.64 ± 0.08	1.62 ± 0.08	0.49
Weight (kg)	69 ± 11	63 ± 12	0.16
BMI (kg/m^2^)	25 (22, 27)	23 (21, 26)	0.27
Underwent IVF (# of patients)	9 (56%)	2 (12%)	0.01
CKD stage at the time of the latest MRI			0.008
Stage G1	6 (37%)	14 (87%)	
Stage G2	8 (50%)	2 (13%)	
Stage G3a	2 (13%)	0 (0%)	
Stage G3b	0 (0%)	0 (0%)	
Aspartate Aminotransferase (mg/dL)	19 (17, 22)	24 (19, 27)	0.16
Alanine Aminotransferase (mg/dL)	15 (12, 19)	17 (14, 30)	0.26
Gravida number	2 (2, 3)	2 (1, 2)	0.52
Scans per study period			
During a period including pregnancy	42	41	N/A
During a period of non-pregnancy	60	122	N/A
Interval (days)			
Between MRI exams	693 (393, 906)	330 (180, 526)	<0.001
During the period including pregnancy(ies)	810 (675, 1092 *)	715 (480, 1219)	0.52
During the period of non-pregnancy	578 (320, 848)	259 (145, 439)	<0.001
Between the nearest pre-pregnancy MRI and delivery	489 (325, 790)	493 (375, 774)	0.99
Between delivery and the nearest post-delivery MRI	254 (173, 399)	266 (95, 393)	0.51

* The longest 3 periods included multiple pregnancies.

**Table 2:** Annual growth rates of liver and kidney volumes in female ADPKD patients (*n* = 16) versus non-ADPKD control patients (*n* = 16), and liver cyst and kidney cyst volume in ADPKD patients (*n* = 16) during periods of pregnancy and non-pregnancy.

		Annual Growth Rate (%/yr)		
		During a Period Including Pregnancy	During Non-Pregnancy Period	*p*-Value
Liver volume	ADPKD	6 [2, 7]	0.3 [−0.4, 2]	0.04
	Non-ADPKD	3 (1, 10)	1 (−2, 3)	0.06
	*p*-value	0.78	0.93	
Total kidney volume	ADPKD	6 [4, 8]	3 [0.8, 5]	0.14
	Non-ADPKD	0.1 (−0.8, 2)	0.5 (−0.4, 1)	0.95
	*p*-value	0.002	0.11	
Liver cyst volume	ADPKD	34 ± 16	23 ± 17	0.005
Kidney cyst volume	ADPKD	12 ± 11	4 ± 9	0.05

**Table 3:** Effect of pregnancy order on annual growth rate of liver, kidney, and cyst volume in 16 female ADPKD subjects (total of 24 pregnancies). Note that gravida has no statistically significant effect on the liver, kidney or cyst growth rate during a period including pregnancy.

Annual Growth Rate (%/yr)	1st Pregnancy (*n* = 11)	2nd Pregnancy (*n* = 7 *)	≥3rd Pregnancy (*n* = 6 **)	*p*-Value
Liver volume	5 ± 6	4 ± 6	9 ± 5	0.21
Liver cyst volume	38 ± 17	36 ± 19	20 ± 15	0.11
Total kidney volume	6 ± 7	6 ± 6	5 ± 7	0.92
Kidney cyst volume	11 ± 11	15 ± 12	12 ± 8	0.75

* One of the cases including the growth rate of both the first and second pregnancies. ** One of the cases including the growth rate of both the second and third pregnancies.

**Table 4:** Cumulative effect of prior pregnancies on non-pregnancy annual growth rate of liver, total kidney volume, and cyst volume in female ADPKD patients. Growth rates during non-pregnancy periods are shown relative to pregnancy order (before the 1st pregnancy, after the 1st, after 2nd, and after ≥3rd pregnancies). A total of 21 non-pregnancy periods were analyzed, showing no cumulative effect of having multiple pregnancies on the liver, kidney and cyst growth rates during periods without pregnancy.

Annual Growth Rate (%/yr)	Pre 1st Pregnancy (*n* = 9)	Post 1st Pregnancy (*n* = 4)	Post 2nd Pregnancy (*n* = 3)	Post ≥ 3rd Pregnancies (*n* = 5)	*p*-Value
Liver volume	−0.3 (−1, 1)	1 ± 2	4 ± 6	0.3 ± 2	0.52
Liver cyst volume	37 ± 29	20 ± 20	13 ± 9	15 ± 9	0.13
Total kidney volume	4 ± 4	8 ± 4	2 ± 3	−0.3 ± 7	0.10
Kidney cyst volume	2 ± 7	15 ± 12	8 ± 20	10 ± 9	0.22

**Table 5:** Comparison of the annual volume growth rate of liver, liver cysts, kidneys, and kidney cysts in ADPKD subjects during a period with IVF-induced pregnancy versus during a period with a naturally conceived pregnancy.

Annual Growth Rate (%/yr)	IVF-Induced Pregnancy * (5 Pregnancies from 4 ADPKD Subjects)	Naturally Conceived Pregnancy (21 Pregnancies from 12 ADPKD Subjects)	*p*-Value
Liver volume			
During a period including pregnancy	3 ± 7	6 ± 6	0.33
During non-pregnancy	0.1 (−1.3, 7)	0.2 (−0.4, 3)	0.75
Liver cyst volume			
During a period including pregnancy	38 ± 22	29 ± 17	0.53
During non-pregnancy	7 (0.8, 27)	21 (10, 31)	0.37
Total kidney volume			
During a period including pregnancy	4 ± 6	6 ± 7	0.41
During non-pregnancy	5 ± 6	3 ± 5	0.63
Total kidney cyst volume			
During a period including pregnancy	9 (2, 12)	12 (8, 16)	0.41
During non-pregnancy	2 ± 13	8 ± 11	0.27

* The period of IVF was included within the during pregnancy period.

**Table 6:** Annual growth rate of liver and TKV during pregnancy and non-pregnancy periods in non-ADPKD control patients.

	Annual Growth Rate (%/yr)		
	During a Period Including Pregnancy (*n* = 16)	During Non-Pregnancy Period (*n* = 16)	*p*-Value
Liver volume	3 (1, 10)	1 (−2, 3)	0.06
Total kidney volume	0.1 (−0.8, 2)	0.5 (−0.4, 1)	0.95

## Data Availability

The data used for this study are available and can be shared with a data sharing agreement upon request from the corresponding author after the de-identification of patients’ information.

## Supplementary Materials

The following supporting information can be downloaded at: https://www.mdpi.com/article/10.3390/jcm14113688/s1, Supplemental Table S1. Effect of pregnancy on liver and kidney parameters in ADPKD women and control subjects, women without ADPKD. Supplemental Table 2. Effect of pregnancy on liver and kidney parameters in ADPKD women before any pregnancy and after pregnancy. Supplemental Table S3. The difference between ADPKD patients with and without an increase in hepatic cyst growth during pregnancy. Supplemental Table S4. The difference between ADPKD patients with and without an increase in kidney cyst growth during pregnancy.

## Abbreviations

The following abbreviations are used in this manuscript:

ADPKD	Autosomal dominant polycystic kidney disease
TKV	Total kidney volume
Ht-TKV	Height-adjusted total kidney volume
Ht-liver cyst volume	Height-adjusted liver cyst volume
Ht-total kidney cyst volume	Height-adjusted total kidney cyst volume
MRI	Magnetic Resonance Imaging
IVF	In vitro fertilization
MIC	Mayo imaging classification
HIPAA	Health Insurance Portability and Accountability Act
BMI	Body mass index
SSFSE	Single shot fast spin echo
SSFP	Steady-state free precession
SD	Standard deviation
PLD	Polycystic liver disease
LFT	Liver function test
GA	Gestational age
