# Supplementary material for: Effects of Pregnancy on Liver and Kidney Cyst Growth Rates in Autosomal Dominant Polycystic Kidney Disease: A Pilot Study
Source: J Clin Med. 2025 May 24;14(11):3688. doi: 10.3390/jcm14113688 (PMC12156408; doi:10.3390/jcm14113688)
Supplement: Supplementary file 1 [file jcm-14-03688-s001.zip › jcm-3590994-supplementary.pdf]

Supplemental materials

Supplemental Table S1. Effect of pregnancy on liver and kidney parameters in ADPKD women and control subjects, women without ADPKD.

	ADPKD (*n*=16)	Control non-ADPKD (*n*=16)	p-value
**Liver volume**			
Pre-pregnancy (ml)	1594 (1377, 1882)	1166 (1077, 1315)	<0.001
Post-pregnancy (ml)	1641 (1372, 2387)	1491 (1335, 1774)	0.02
Pre-pregnancy growth rate for 8 ADPKD and 8 non-ADPKD (%/yr)	0 [-0.7, 0.9]	2 (-1, 5)	0.46
During a period including pregnancy growth rate - 16 subjects (%/yr)	6 [2,7]	3 (1, 10)	0.78
Post-pregnancy growth rate - 10 ADPKD and 12 non-ADPKD (%/yr)	0.3 [-0.3, 3]	-0.3 (-4, 3)	0.22
**Height-adjusted liver volume (ml/m)**			
Pre-pregnancy MRI	571 [527, 732]	432 (403, 526)	<0.001
Post-pregnancy MRI	595 [506, 946]	533 (485, 739)	0.03
**Liver parenchymal growth rate (%/yr)**			
During a period including pregnancy	4 (2, 5)	3 (1, 10)	0.78
During the non-pregnancy period	-1 (-1, 0.3)	1 (-2, 3)	0.42
**Total kidney volume (ml)**			
Pre-pregnancy MRI	710 [523, 925]	311 (279, 321)	<0.001
Post-pregnancy MRI	812 [602, 1751]	335 (300, 361)	<0.001
Pre-pregnancy growth rate - 8 ADPKD and 8 non-ADPKD (%/yr)	4 ± 4	2 ± 6	0.57
During a period including pregnancy growth rate - 16 subjects (%/yr)	6 [4, 8]	0.1 (-0.8, 2)	0.002
Post-pregnancy growth rate - 10 ADPKD and 12 non-ADPKD (%/yr)	3 [1, 7]	0.1 (-0.6, 1)	0.05
**Height-adjusted total kidney volume (ml/m)**			
Pre-pregnancy	264 [199, 361]	110 (107, 123)	<0.001
Post-pregnancy	304 [224, 730]	124 (117, 135)	<0.001
**Total kidney parenchymal growth rate (%/yr)**			
During a period including pregnancy	3 [1,5]	0.1 (-0.8, 2)	0.1
During the non-pregnancy period	1 [-0.7, 4]	0.5 (-0.4, 1)	0.56

Supplemental Table S2. Effect of pregnancy on liver and kidney parameters in ADPKD women before any pregnancy and after pregnancy

	Before Pregnancy (*n*=8)	After Pregnancy (*n*=10)	p-value
**Liver**			
Liver growth rate (%/yr)	-0.3 [-1, 0.9]	0.3 [-0.3, 3]	0.18
Liver cyst growth rate (%/yr)	39 ± 30	16 ± 12	0.02
**Kidney**			
TKV growth rate (%/yr)	4 ± 4	3 ± 6	0.84
Total kidney cyst volume growth rate (%/yr)	2 ± 8	11 ± 12	0.07

Supplemental Table S3. The difference between ADPKD patients with and without an increase in hepatic cyst growth during pregnancy.

	Hepatic Cyst Growth Rate During Pregnancy		p-value
	**Increased**	**Not increased**	
**Number of patients**	14	2	
**Age at 1st pregnancy (yr)**	34 (33, 36)	34*	1
**Height (m)**	1.65 ± 0.08	1.54 ± 0.03	0.09
**Weight (kg)**	68 ± 11	71 ± 11	0.7
**BMI (kg/m²)**	25 ± 4	29 ± 4	0.16
**Ht-TKV (ml/m)**	330 (236, 541)	318 (312, 323)	0.93
**Ht-liver volume (ml/m)**	627 (579, 775)	2238 (1414, 3061)	0.5
**Ht-liver cyst volume**	26 (8, 122)	1380 (703, 2056)	0.33
**GA at delivery (days)**	263 (237, 277)	90 (90, 136)	0.16
**Newborn weight (grams)**	3033 (2438, 3605)	2224*	0.29
**Preeclampsia**	4 (29%)	0 (0%)	0.38
**Comorbidity**	8 (57%)	1 (50%)	0.85
**Aspirin use**	9 (64%)	2 (100%)	0.3
**IVF**	7 (50%)	2 (100%)	0.18
**Proteinuria**	0 (0%)	0 (0%)	1
**Urine specific gravity**	1.005 (1.003, 1.008)	1.015 (1.014, 1.015)	0.06
**CKD stage**			0.06
Stage G1	6 (43%)	0 (0%)	
Stage G2	7 (50%)	1 (50%)	
Stage G3a	1 (7%)	1 (50%)	
Stage G3b	0 (0%)	0 (0%)	

*Data available for one case only.

Supplemental Table S4. The difference between ADPKD patients with and without an increase in kidney cyst growth during pregnancy.

	**Kidney cyst growth during pregnancy**		**p-value**
	**Increased**	**Not increased**	
**Number of patients**	12	4	
**Age at 1st pregnancy (yr)**	34 ± 2	34 ± 2	0.95
**Height (m)**	1.65 ± 0.08	1.58 ± 0.05	0.15
**Weight (kg)**	69 ± 11	68 ± 9	0.97
**BMI (kg/m^2)**	25 ± 4	27 ± 3	0.25
**Ht-TKV (ml/m)**	307 (226, 535)	334 (323, 482)	0.45
**Ht-liver volume (ml/m)**	627 (583, 782)	691 (560, 1566)	0.95
**Ht-kidney cyst volume (ml/m)**	120 (60, 311)	166 (136, 311)	0.45
**GA at delivery (days)**	258 (237, 277)	272 (269, 275)	0.29
**Newborn weight (g)**	3033 (2722, 3447)	2307 (2224, 2695)	0.25
**Preeclampsia**	0 (0%)	0 (0%)	1
**Comorbidity**	6 (50%)	3 (75%)	0.38
**Aspirin use**	7 (58%)	4 (100%)	0.12
**Proteinuria**	0 (0%)	0 (0%)	1
**Urine specific gravity**	1.005 (1.003, 1.008)	1.01 (1.005, 1.014)	0.39
**CKD stage**			0.005
G1	6 (50%)	0 (0%)	
G2	6 (50%)	2 (50%)	
G3a	0 (0%)	2 (50%)	
G3b	0 (0%)	0 (0%)	
